# Supplementary material for: Correlative X-ray imaging and fluorescence microscopy in hydrated environments
Source: J Synchrotron Radiat. 2026 Oct 12;33(Pt 6):1779–93. doi: 10.1107/S1600577526009513 (PMC13753531; doi:10.1107/S1600577526009513)
Supplement: Supplementary file 1 [file s-33-01779-sup1.pdf]

JOURNAL OF SYNCHROTRON RADIATION

Volume 33 (2026)

Supporting information for article:

Correlative X-ray imaging and fluorescence microscopy in hydrated environments

Mangalika Sinha, Boram Yu, Rita Mendes Da Silva, Ulrike Rölleke, Peter Luley, Malte Tiburcy, Wolfram-Hubertus Zimmermann, Manfred Burghammer and Sarah Köster

Table S1: List of components used to construct the home-built fluorescence microscope set-up, sorted along the light path

**Component**	**Model**	**Company**	**Specifications**
LED light source	X-Cite Xylis II, XT730L	Excelitas Technologies Pittsburgh, PA, USA	Emission spectrum: 360 nm – 770 nm
Light guide	–	Excelitas Technologies	Diameter: 3 mm;<br>Length: 1.5 m
Aspheric collimating lens	ACL2520U-A	Thorlabs, Newton, NJ, USA	Focal length $f = 30$ mm;<br>Numerical aperture NA = 0.6
Kinematic cage system	DFM1T1	Thorlabs	30 mm cube for filter set
20× objective	UCPLFLN20X	Evident, Hamburg, Germany	NA = 0.7;<br>Working distance WD = 0.8 – 1.8 mm;<br>Adjustable cover glass correction 0 – 1.6 mm
GFP filter set	L70-450GF	AHF, Tübingen, Germany	Excitation filter: 460/50 nm;<br>Dichroic mirror: 495 nm;<br>Emission filter: 525/50 nm
YFP filter set	F36-529	AHF	Excitation filter: 504/24 nm;<br>Dichroic mirror: 521 nm;<br>Emission filter: 539/27 nm
Tube lens	TTL 180A	Thorlabs	$f = 180$ mm;<br>WD = 133.5 mm;<br>anti reflection coating: 350 - 700
Camera	pco.pixelfly 1.4 M-USB	Excelitas Technologies	1392 × 1040 pixels;<br>pixel size: 6.45 μm × 6.45 μm

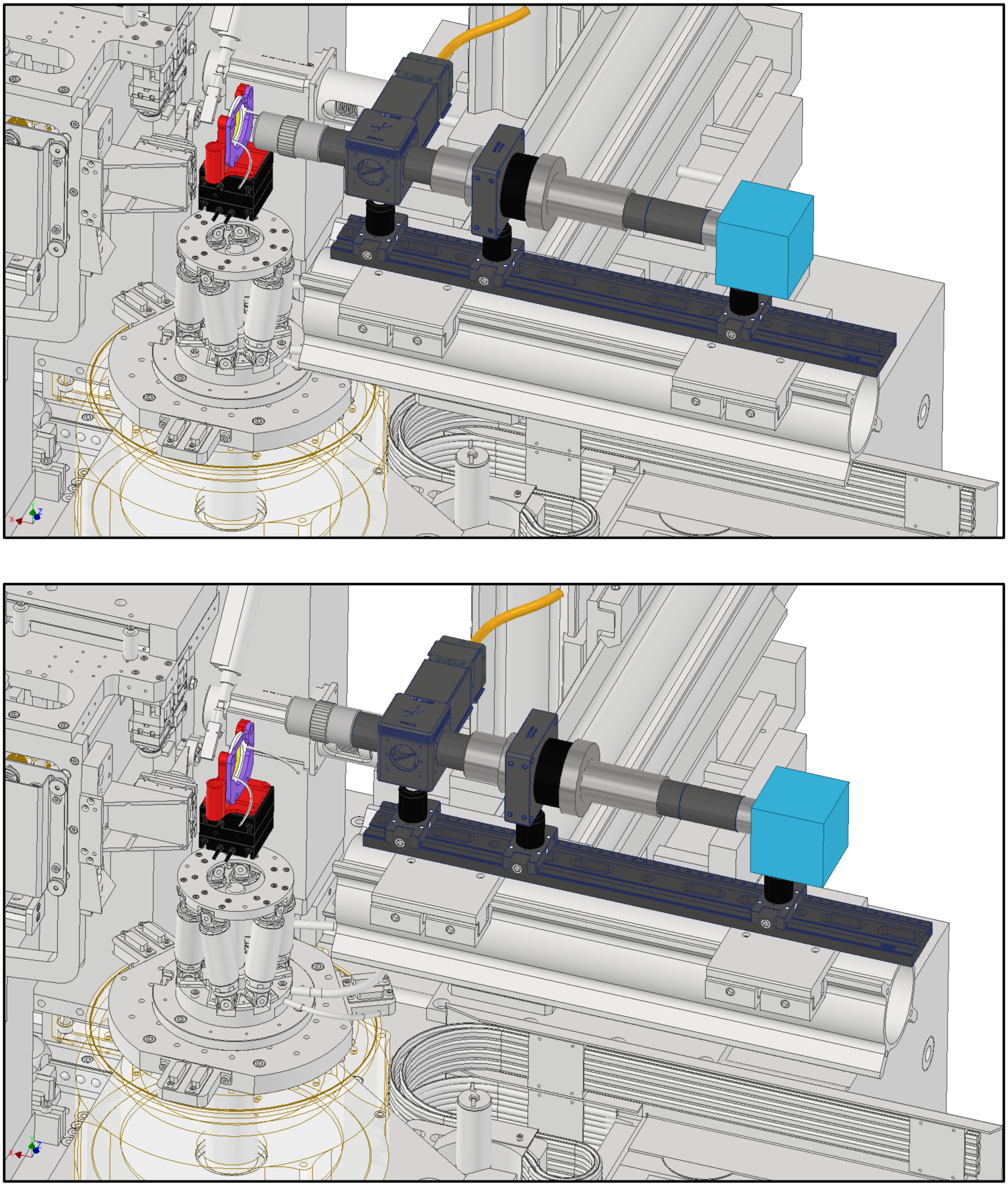

Figure S1: CAD drawings of the fluorescence microscope set-up. (Top) Moved into the X-ray beam path and, therefore, aligned with the microfluidics chamber for the collection of fluorescence images of the sample. (Bottom) Moved out of the X-ray beam path. The translation range is 125 cm.

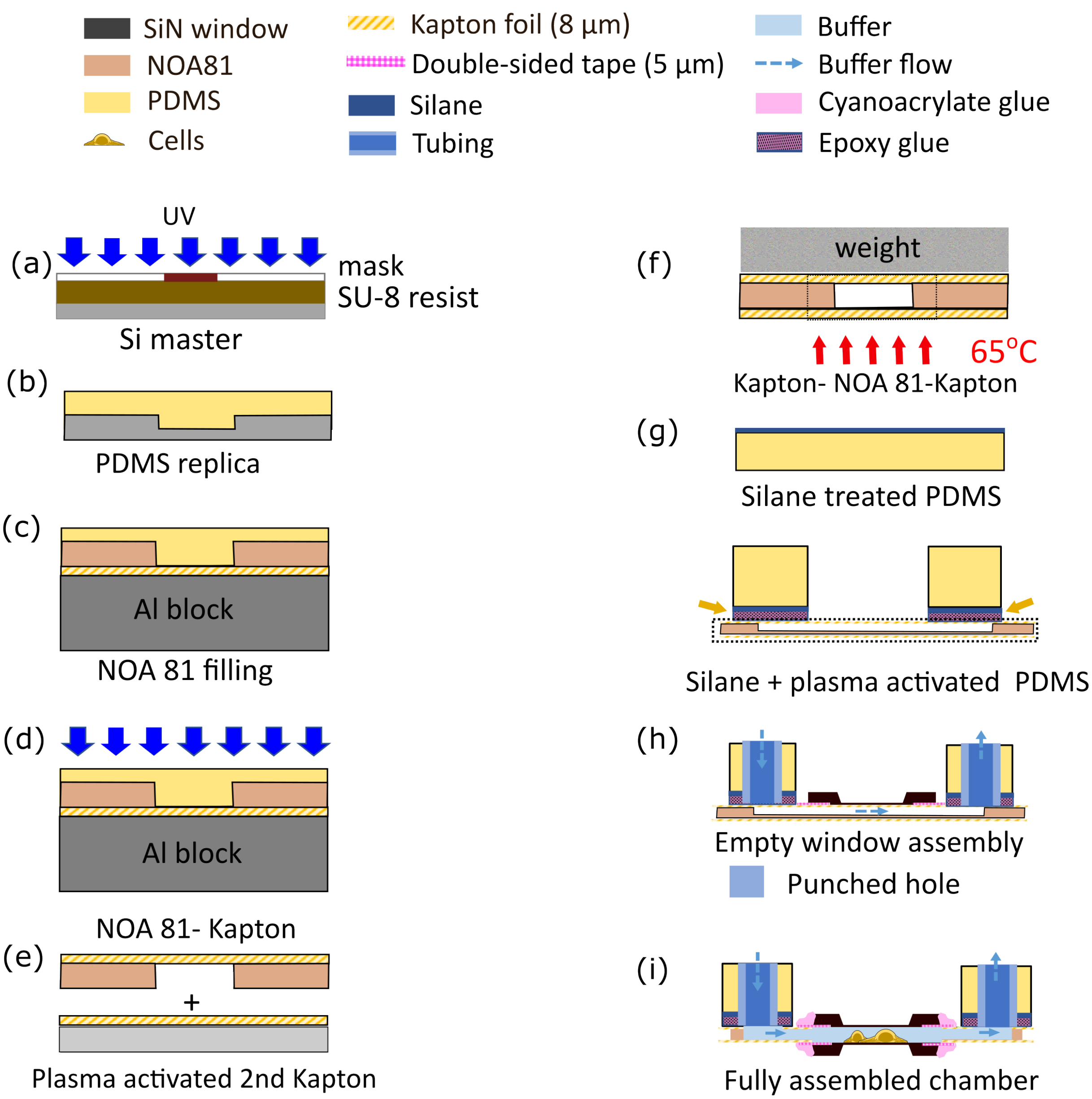

Figure S2: Step-wise schematic of the fabrication workflow of the microfluidic chamber. (a) Photolithography to define the channel pattern by SU-8 photoresist on a silicon wafer. (b) PDMS replica molding from the silicon master. (c) NOA 81 replica molding on Kapton foil by capillary filling of the PDMS replica placed on an aluminum support and (d) UV curing of the NOA 81 forming a stable NOA 81-Kapton bond. (e) Sealing of the cured NOA 81-Kapton structure with a second, plasma activated Kapton foil. (f) Baking of the Kapton-NOA 81-Kapton structure (referred to as *device*) while being pressed down by a small weight. The dotted box indicates the channel region of the device. (g) Preparation of PDMS for device support. Bonding between the device and PDMS is achieved by silanization, plasma activation and applying epoxy glue. The activated side of PDMS support is indicated by orange arrows. (h) Punching holes in the center of device and the inlet/outlet. An empty Si_3N_4 window is attached to the center of the device with double-sided tape. (i) Fully assembled microfluidic chamber.

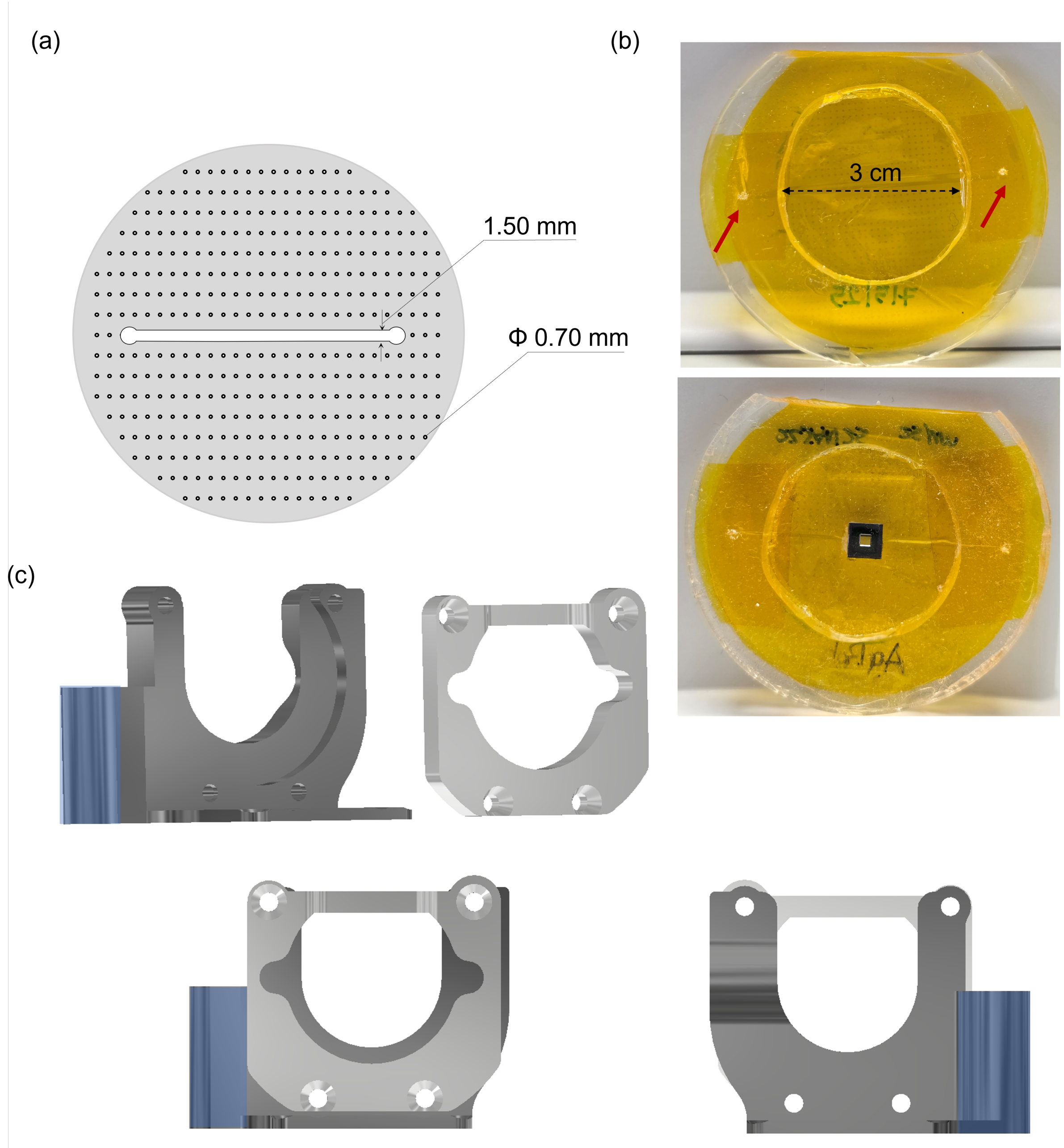

Figure S3: (a) Photomask design. The outer diameter is 2 inches. The channel structure is centered on the mask; around the channel structure, we add periodic cylinders with a diameter of 700 μm and 1000 μm center-to-center distance as support structures to facilitate capillary filling (Supplementary Fig. S2c). (b) (Top) Photograph of the Kapton–NOA–Kapton device assembled on a PDMS support, forming the base of the microfluidic flow chamber. The inlet and outlet are indicated by red arrows. (Bottom) Image of the device after attaching an empty Si_3N_4 window. (c) CAD drawings of the chamber holder. (Top) Separate drawings of the two components. (Bottom) Front and back views of the fully assembled holder. The front faces the inlet and outlet tubing side of the ring–shaped PDMS support and is oriented towards the objective of the fluorescence microscope. The back view faces the incoming X-ray beam. The reservoir for collecting buffer from the outlet side is shown in blue.

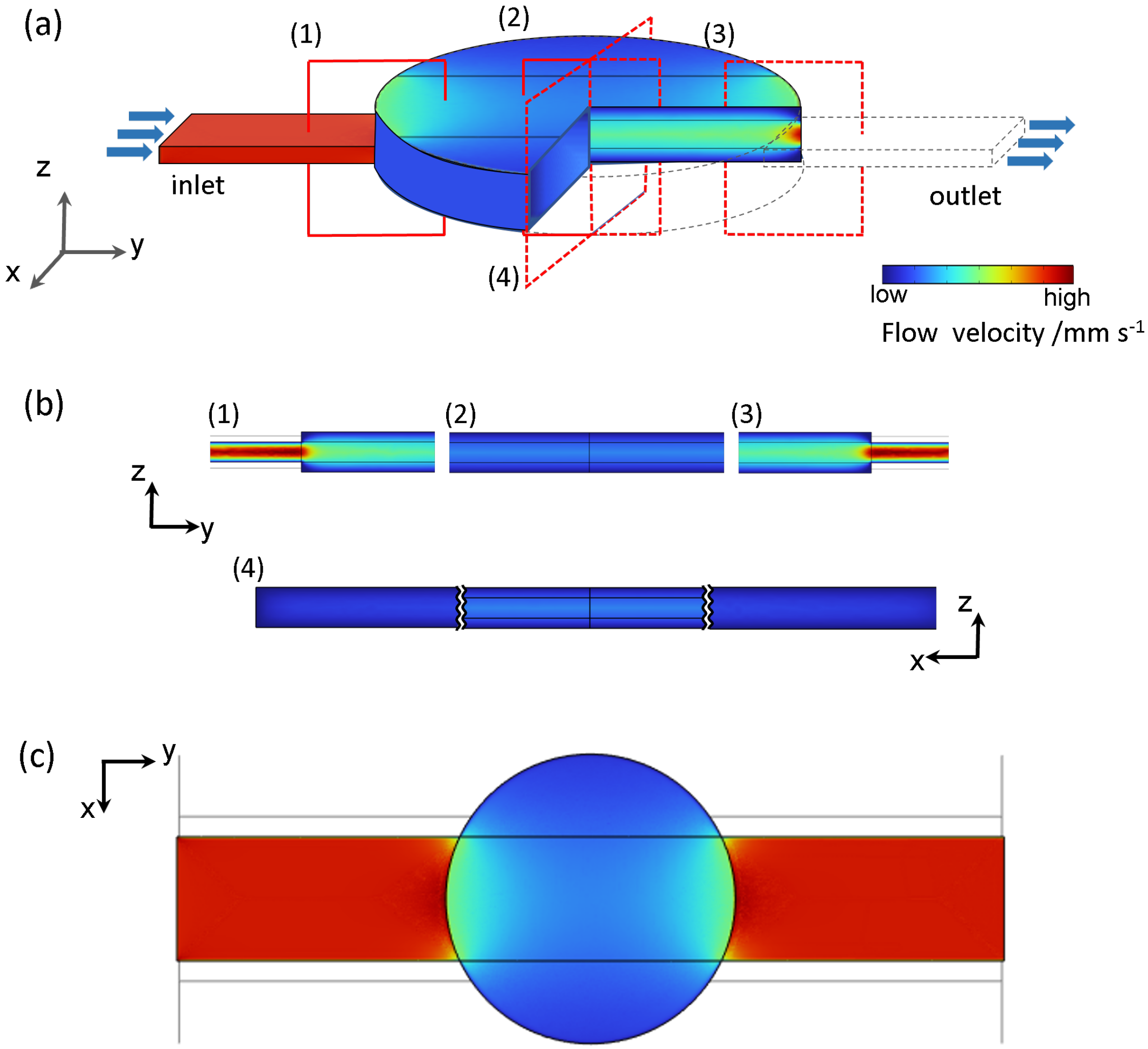

Figure S4: Finite element method (FEM) simulation of the flow conditions within the microfluidic chamber (COMSOL multiphysics 6.2). (a) 3D representation of the flow velocity in mm/s within the straight microchannel and observation region in the center defined by a "sandwich" of two Si_3N_4 windows. Arrows indicate the flow direction. The color map represents the simulated flow velocity, highlighting reduced flow velocity inside the observation region compared to the narrow channels. (b) Longitudinal cross-sections showing the evolution of the flow velocity from inlet to outlet for four specific regions indicated as (1) to (4) in panel a. Regions (2) and (4) correspond to the membrane region of the Si_3N_4 window, where fluorescence microscopy and scanning SAXS measurements are performed. (c) Top view illustrating the spatial distribution of flow velocities across the observation region–channel interface.

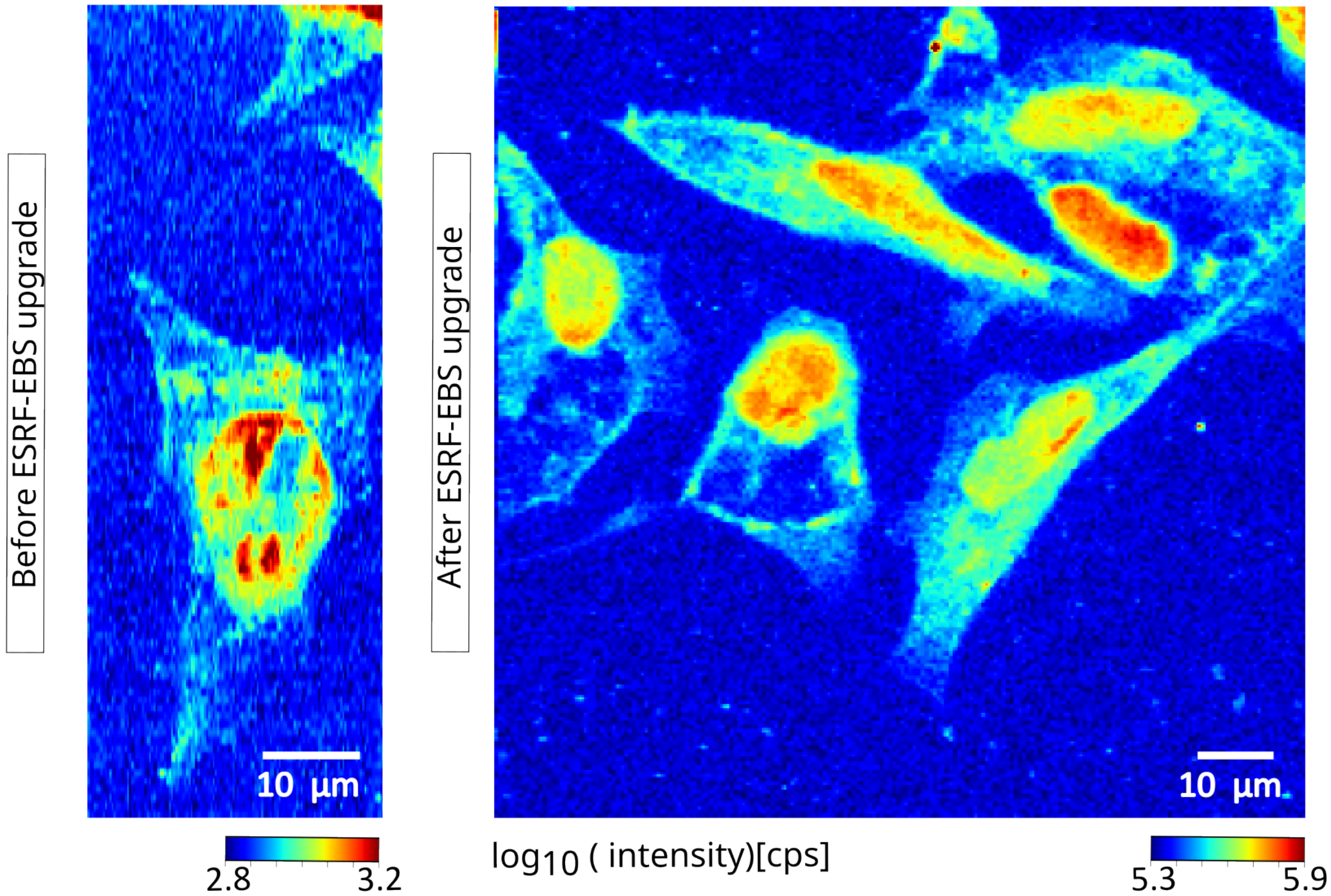

Figure S5: X-ray dark-field images of fixed-hydrated SW13 cells before and after the "Extremely Brilliant Source" (EBS) upgrade at ESRF. The water layer thickness of the microfluidic chamber before the upgrade was 200 μm, whereas the newly designed microfluidic chamber compatible with both optical imaging and fast scanning SAXS modalities has a water layer thickness of 60± 10 μm. The scanning parameters for the respective measurements are listed in Supplementary Table S2. The q-range considered for calculating the data before the upgrade is (0.175–0.262) nm^{-1}, whereas for the present beamtime it is (0.19–0.39) nm^{-1}, corresponding to the optimization described in (Yu *et al.*, 2026).

Table S2: Parameters for fast scanning SAXS before and after the EBS upgrade at ESRF

**Parameters**	**Pre upgrade**	**Post upgrade**
Photon energy [keV]	14.92	15.00
Photon flux [ph/s]	8.7×10^9	1.2×10^{12}
Exposure time [s]	0.5	0.002
Step size ($\Delta_{y,z}$ [μm^2])	0.3×1	0.5×0.5
Dose per scan point [Gy]	5.3×10^6	6.3×10^6
Scattering contrast $\left(\frac{I_{\text{cell}}-I_{\text{bkg}}}{I_{\text{bkg}}}\right)$	1.5	3.0

Table S3: Calculated flow velocities in the channel and the observation region for different flow rates

**Location**	**Width (μm)**	**Height (μm)**	**Cross-sectional area (m^2)**	**Flow rate (μL/h)**	**Flow velocity (mm/s)**
Observation region	3500	60	2.1×10^{-7}	20	0.026
				50	0.063
				100	0.132
				200	0.263
Channel	1500	25	2.25×10^{-8}	20	0.246
				50	0.62
				100	1.24
				200	2.48

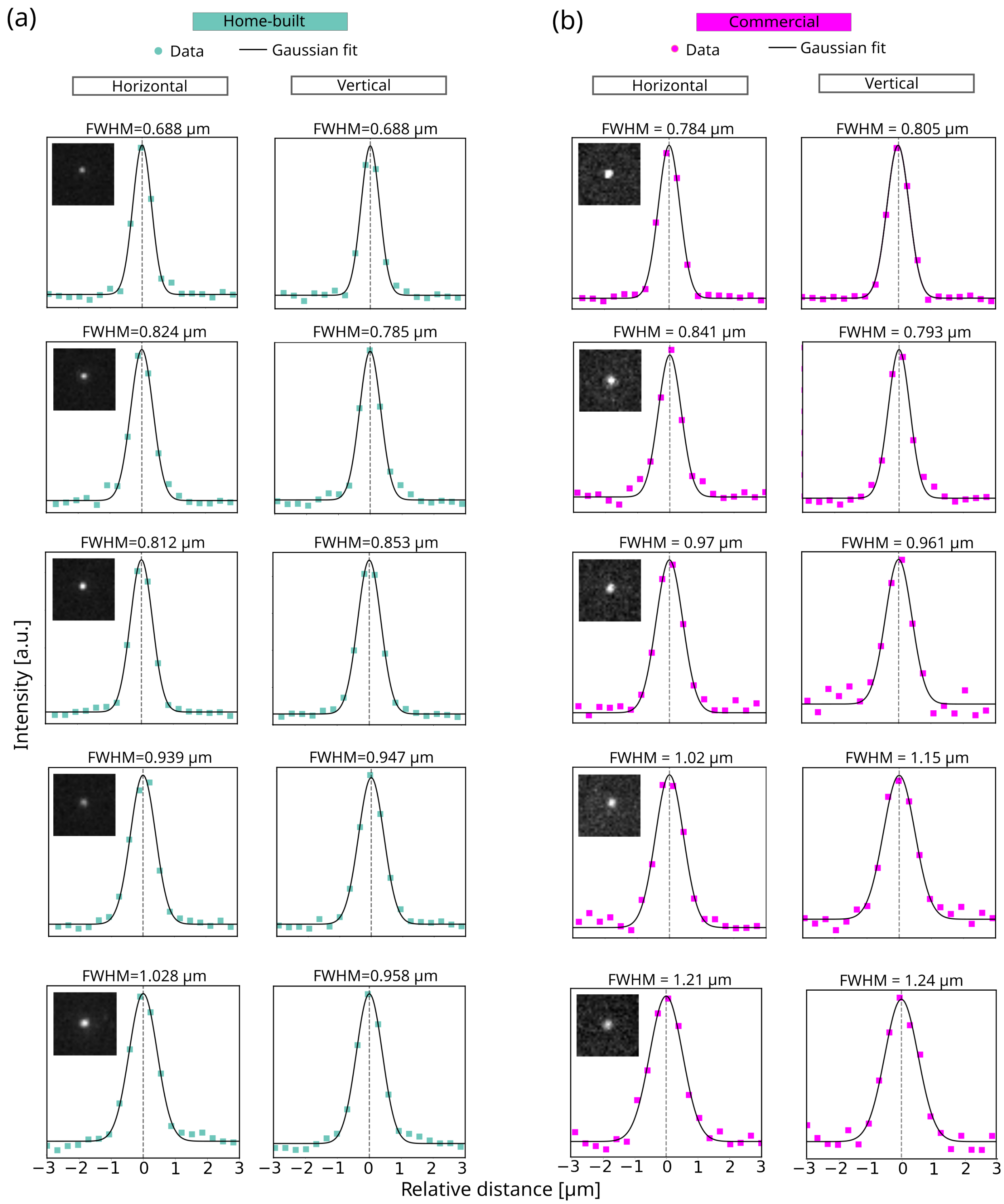

Figure S6: Comparison of lateral point spread functions (PSFs) obtained using the home-built and commercial fluorescence microscopes. a) Representative PSFs from individual fluorescent beads imaged using the home-built (left, cyan) and b) the commercial (right, magenta) microscope setups. Each inset displays the corresponding bead image and the plots show line intensity profiles (colored circles) along the horizontal (x) and vertical (y) axes through the bead centroids with Gaussian fits (black solid lines).

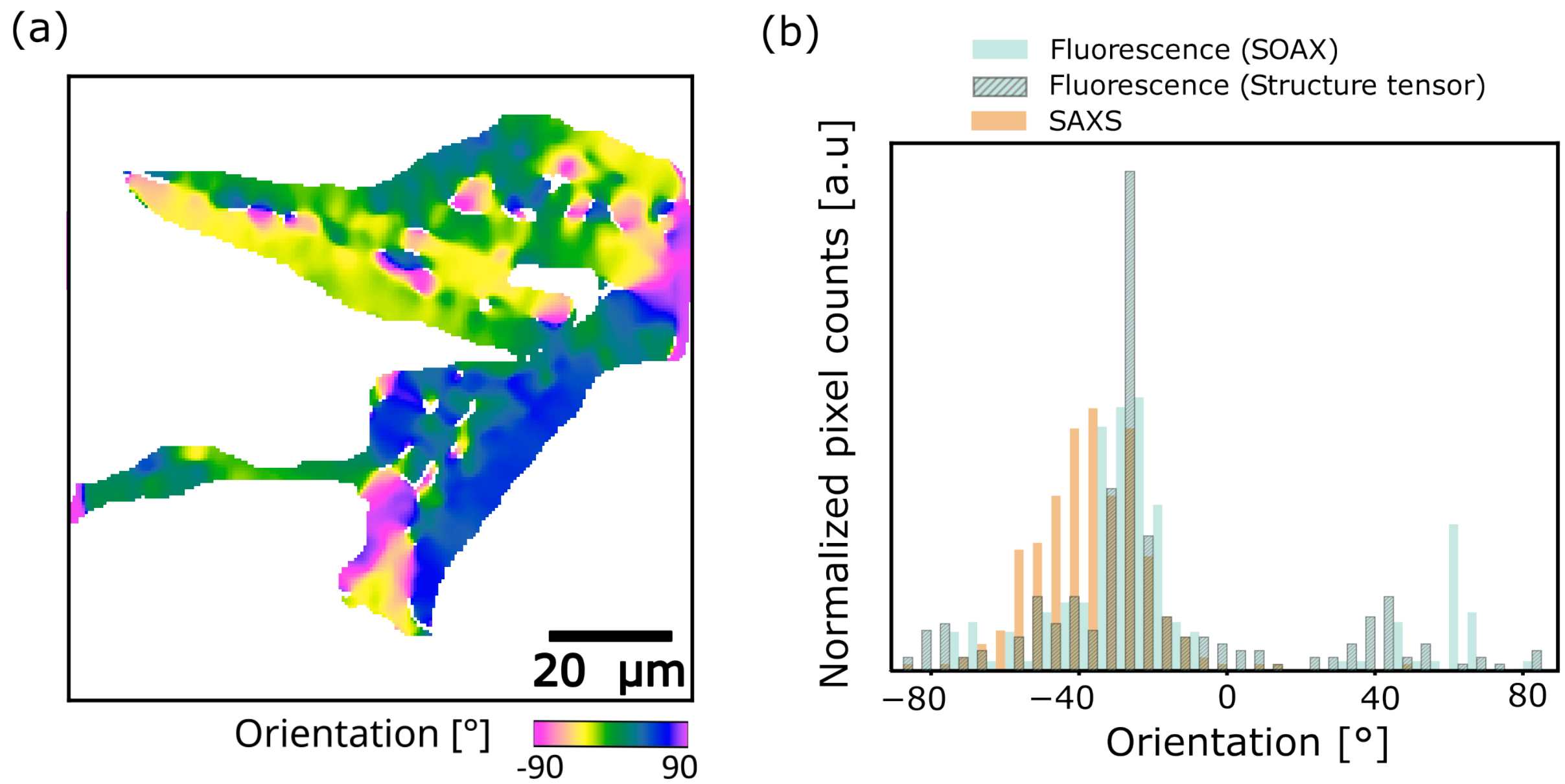

Figure S7: (a) Orientation map derived from the fluorescence image of keratin networks in SW-13 cells (see Fig. 4a in the main text) using the structure tensor method. (b) Comparison of the orientation histograms obtained from SAXS and fluorescence analysis using SOAX (as shown in the main text) and the structure tensor method. Only the pixels within the white outlined region of Fig. 4d of the main text are included in this analysis.

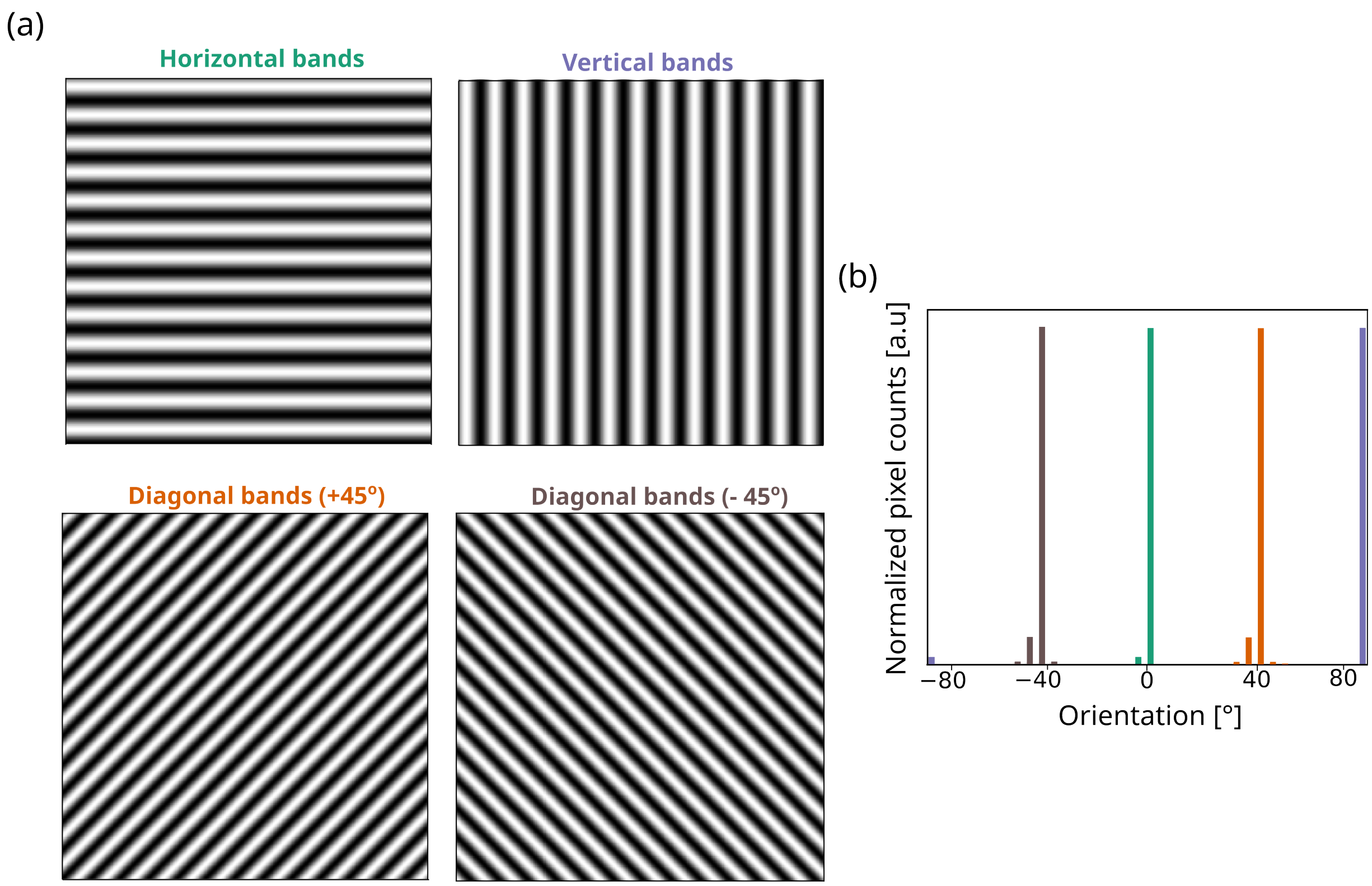

Figure S8: Application of the structure tensor method to synthetic data. (a) Grayscale images containing horizontal, vertical and diagonal (+45° and -45°) stripe patterns. (b) The corresponding orientation histograms show distinct peaks at the expected angles, demonstrating that the structure tensor analysis reliably captures dominant alignment across different orientations.
